# Acetic Acid and Ammonium Persulfate Pre-Treated Copper Foil for the Improvement of Graphene Quality, Sensitivity and Specificity of Hall Effect Label-Free DNA Hybridization Detection

**DOI:** 10.3390/ma13071784

**Published:** 2020-04-10

**Authors:** Naiyuan Cui, Fei Wang, Hanyuan Ding

**Affiliations:** Ministry of Education Key Laboratory for Non-Equilibrium Synthesis and Modulation of Condensed Matter, School of Science, Xi’an Jiaotong University, Xi’an 710049, China; haotaiyuan1111@163.com

**Keywords:** graphene-based biosensors, hall effect measurement, DNA hybridization, acetic acid pre-treated copper foil, ammonium persulfate pre-treated copper foil

## Abstract

The capability of graphene-based biosensors used to detect biomolecules, such as DNA and cancer marker, is enormously affected by the quality of graphene. In this work, high quality and cleanness graphene were obtained by CVD based on acetic acid (AA) and ammonium persulfate (AP) pretreated copper foil substrate. Hall effect devices were made by three kinds of graphene which were fabricated by CVD using no-treated copper foil, AA pre-treated copper foil and AP pre-treated copper foil. Hall effect devices made of AA pre-treated copper foil CVD graphene and AP pre-treated copper foil CVD graphene can both enhance the sensitivity of graphene-based biosensors for DNA recognition, but the AA pre-treated copper foil CVD graphene improves more (≈4 times). This may be related to the secondary oxidation of AP pre-treated copper foil in the air due to the strong corrosion of ammonium persulfate, which leads to the quality decrease of graphene comparing to acetic acid. Our research provides an efficient method to improve the sensitivity of graphene-based biosensors for DNA recognition and investigates an effect of copper foil oxidation on the growth graphene.

## 1. Introduction

Graphene, a kind of 2D material, has attracted great attention since it was first peeled off in 2004 from graphite via mechanical exfoliation method [1]. Graphene possesses many excellent properties, including: ultrahigh high electron mobility (15,000 cm^2^/V s), good mechanical strength, lowest electrical resistivity (≈8–10 Ωm), high surface area (theoretically 2630 m^2^/g) and good biocompatibility for biomolecules and macromolecules [2,3,4].

Owing to all these great properties, graphene has important research value as a biosensing application [5,6,7,8,9,10,11]. So far, graphene sensors have been used for the detection of gas, pressure, strain, heavy metal ions and biological substances, etc. [12,13,14,15,16] Among all these sensors, biosensors are widely investigated due to the concentration of human health monitoring for the detection of tumor markers, dopamine, biological ions in human body, glucose, proteins and nucleotides. In order to avoid genetic diseases and chromosome disease, the detection of deoxyribonucleic acid (DNA) sequence is of significant importance in genetic disease detection and genetic engineering. Many methods have been investigated to identify DNA targets such as electrochemical, electrical detection methods and fluorescent based on graphene. Recently, label-free electrical techniques have caught much attention due to the advantages of simplicity, time saving and cost-effectiveness [17,18,19,20]. Unlike electrochemical and fluorescent, electrical techniques do not need extra fluorescent or electrochemical tags [21,22]. Several kinds of graphene have already been fabricated as important sensing materials to make label-free sensors for the detection of DNA, including mechanically exfoliated graphene [23,24], graphene oxide (GO) [25,26] and chemical vapor deposition (CVD) graphene [27,28,29,30]. As we know, the sensitivity of sensors greatly relies on the layer number, lateral size and the amount of oxygen-containing functional groups on the surface, which is difficult to control for the graphene oxide (GO). As for mechanically exfoliated graphene, the graphene layers, the morphology and the quality of graphene are uncontrollable and can’t be guaranteed. In contrast, chemical vapor deposition (CVD) method has risen rapidly in the last few years, which makes it possible to fabricate graphene with high quality, large area, controllable structure and layers. This kind of graphene is suitable candidate for the fabrication of sensors. The principle of CVD is to crack carbon sources into carbon atoms at high temperatures, which are then deposited on the surface of the transition metal catalyst to form graphene. The most commonly used catalyst is copper foil, whose surface condition is closely related to the quality of graphene. Commercial Copper foil is produced by a calendaring process and the surface is covered with an oxide layer to prevent oxidation. This kind of oxide layer is harmful to the growth of graphene, and easy to produce wrinkles, amorphous carbon and holes on the surface of graphene. To solve this problem, the methods of surface pre-treatment to remove surface oxide layer have been investigated. Song et al. [31] pre-treated the copper foil surface by chemical polishing to study the conditions for the growth of high-quality graphene. Zhang et al. [32] used copper foil as an electrode to pre-treat the copper foil by electrochemical polishing method to prepare a large area and high-quality single layer. The result showed that under the same conditions for graphene growth, compared to the unelectropolished Cu foil, graphene coverage area on the surface of electropolished Cu foil was larger. Luo et al. [33] firstly combined mechanical polishing with electrochemical polishing to pre-treat the surface of the copper foil to obtain high-quality graphene. However, there are various shortcomings in the mechanical or electrochemical polishing of copper foil. Mechanical polishing tends to introduce extra stress on copper foil, and it is unavoidable to produce scratches on the copper foil surface. The electrochemical polishing process is complicated to carry out, and complex electrolyte systems are required, with high operating requirements and high energy consumption. Compared to the above two methods, pre-treating the copper foil with acid seems to be not only convenient but also easy to operate without producing damage to the copper foil. Kim MS, et al. reported a method to pre-treated Cu copper foil with pre-H_2_ annealing and nitric acid treatment. They synthesized high quality and uniform graphene on Cu by this method which can effectively reduce the impurities on Cu. Raman mapping results showed that 98.3% of the mapped area exhibited monolayer characteristics with I_2D_/I_G_ > 2 compared to 75% monolayer characteristics for that grown on untreated Cu foil [34]. Jeong H, et al. used ammonium persulfate solution combined with gentle ultrasonication (100 W) to remove the impurities on copper foil. This method cannot only remove the impurities on copper foil, but also won’t leave metal ion residue on the cleaned copper substrates, which can help obtain highly uniform monolayer graphene [35]. Senyildiz D, et al. studied the influence of acid pretreatment to control the densities of Si and Ca based impurities on the surface. They investigated the surface coverage and morphology of graphene by scanning electron microscope (SEM) studies, optical microscope (OM) and Raman spectroscopy. Their study showed that acid pre-treatment can effectively remove the surface oxide layer and surface Ca impurities on the copper foil. The coverage and the morphology of the graphene were strongly influenced by the acid pretreatment [36].

In this work, we adopted a convenient and economical way to pre-treat the copper foil surface by chemical etching method attempting to optimize the roughness and cleanliness of copper foil as well as the growth quality of graphene and device sensitivity based on graphene grown on pre-treated copper foil. Before CVD growth, copper foils were immersed in acetic acid and ammonium persulfate separately for 10 s. Three kinds of graphene were used to produce graphene including, non-treated copper foil, AA pre-treated copper foil and AP pre-treated copper foil. After graphene growth ended, these three kinds of graphene were transferred onto SiO_2_/Si substrate for fabrication of Hall effect biosensor by commonly-used PMMA. As shown by microscopy, the surface of AA pre-treated copper foil and the grown graphene were the cleanest and flattest among others. At the same time, AA grown graphene and AP grown graphene could both improve the sensitivity of the biosensors to detect DNA. At the same time, the sensitivity of the Hall effect biosensor fabricated by graphene grown on AA pre-treated copper foil for the detection of DNA was significantly (≈4 times) enhanced compared with that of non-treated graphene grown on pristine copper foil and the specificity of Hall effect biosensor made of graphene grown on AA pre-treated copper foil for the detection of DNA was also greatly enhanced (≈14 times) in comparison to graphene grown on pristine copper foil. This research offers a commercial and easy way to pre-treat the copper foil for the quality improvement of graphene which has a great influence on the sensing performance of the biosensors. The Hall effect biosensor also possesses an excellent sensitivity and specificity, which can effectively distinguish complementary target DNA from one-base mismatched DNA.

## 2. Materials and Methods

### 2.1. Materials and Reagents

Copper foils with 25 µm thickness (purity: 99.8%) for the growth of graphene were obtained from Alfa Aesar Chemical (Shanghai, China) Co. Ltd. Acetic acid, ammonium persulfate (NH_4_S_2_O_8_) were purchased from Sinopharm Chemical Reagent Co. Ltd. (Shanghai, China). Si substrate with 300 nm-thick SiO_2_ coating was bought from Bokete Technology Co. Ltd. (Harbin, China). PMMA as well as Silicone rubber was obtained from Micro Chem Co. Ltd. (MD, USA) and Dow Corning Co. Ltd. (Guangzhou, China) separately. All the other chemical reagents used in this experiment, including PBS buffer solution, probe DNA (5′–AGG–TCG–CCG–CCC–3′), complementary target DNA (3′–TCC–AGC–GGC–GGG–5′) and one base-mismatched DNA (3′–TCC–AGC–GGC–AGG– 5′) were all purchased from Sangon Biotech Co. Ltd. (Shanghai, China).

### 2.2. AA and PA Pretreatment of Copper Foil

Three identical pieces of 5 cm × 5 cm copper foil was firstly sonicated in acetone to remove organic residuals for 10 min and then sonicated in alcohol for another 10 min to remove the acetone on copper foils, followed by sonication in deionized water for 10 min. After that, the copper foils were blown with a nitrogen gun to remove the deionized water. The first piece of copper was left untreated; the second piece was soaked in 0.05 mol/L ammonium persulfate solution for 3 min, then sonicated in deionized water for 5 min followed by blown with a nitrogen gun to remove the deionized water left on it; the third piece was soaked in a 0.05 mol/L acetic acid solution for 3 min, then proceeded the subsequent process similarly with the second copper foil.

### 2.3. Growth of Graphene

Three kinds of graphene were synthesized on three kinds of Cu foil by the CVD method under the same reaction conditions. The Cu foils were firstly heated from 27 °C to 1050 °C in 60 min with a fixed rate. A continuous 20 sccm H_2_ gas flow flowed through the CVD tube, of which the pressure was maintained at 0.34 torr. The furnace temperature was then maintained for 30 min to reduce the native oxide layer. Afterwards, a continuous H_2_/CH_4_ mixed gas flow (8:8 sccm) was introduced for the growth of graphene on Cu foil for 20 min at 1050 °C. The CVD tube was naturally cooled down and the graphene sheet was then transferred onto a target substrate for further investigation.

### 2.4. Characterization of Graphene

The surface topography was characterized using scanning electron microscope (SEM, Sirion200, FEI, USA) and atomic force microscope (AFM, Dimension 3100, Veeco, Plainview, NY, USA). The quality of the graphene was studied by Raman spectroscopy (532 nm He-Ne laser, Renishaw plc, Wotton-under-Edge, UK). The chemical compositions of graphene were analyzed by X-ray photoelectron spectroscopy (XPS, AXIS ULTR DLD, Kratos Analytical, Kyoto, Japan). The surface free energy was analyzed by a surface tension/dynamic contact angle measurement system (OCA20, Data Physics, Berlin, Germany). The room temperature Hall effect measurements were performed with the four-point probe method, which was based on the Van der Pauw theory in our self-built system.

### 2.5. Immobilization of DNA on Graphene

For each detection experiment with the Hall effect biosensor, a 100-μL drop of PBS buffer was added into the testing window and maintained for 2 h to remove the influence of PBS doping on graphene, as well as infiltrate the graphene surface. Then pre-immobilization of probe DNA was maintained on the graphene surface for 12 h as to substrate the influence of probe DNA and enable it to achieve the saturation adsorption on graphene. Rinsing was then done in 1 mL PBS buffer to remove any loosely bound DNA three times. Afterwards, the target or one-base mismatched DNA varying from 1 pM to 100 nM were dropped onto the device in turn for hybridization with probes. For each concentration of target DNA, it took 3 h for hybridization, which was always followed by a rinsing step.

## 3. Results and Discussions

### 3.1. Fabrication of Graphene Hall Effect Biosensor

**Figure 1:**
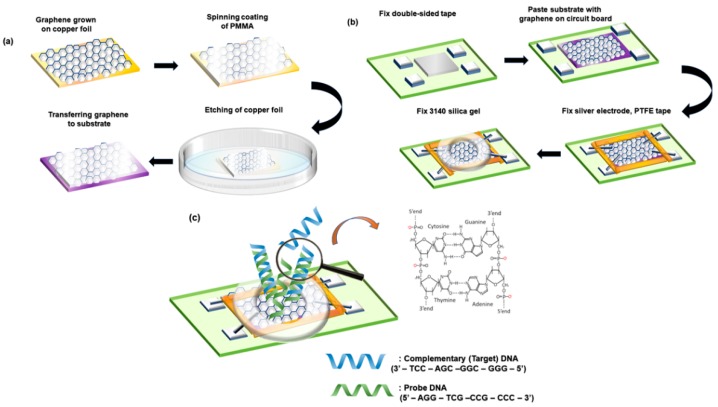
(**a**) Graphene transfer process; (**b**) Hall effect device fabrication process; (**c**) DNA hybridization on graphene.

Figure 1a presents the transfer process adopted in this research in order to transfer the graphene films from the three kinds of copper foils to a SiO_2_/Si substrate separately. In this process, PMMA played a role of the supporting layer. Firstly, a 100 nm-thick PMMA layer was spin-coated on graphene. Then, the subjacent Cu foil was removed through etching in a 0.1 M aqueous (NH_4_)_2_S_2_O_8_ solution for 5 h. After the removal, the graphene/PMMA layer was moved into deionized water three times for cleaning. The layer was then transferred onto the target substrate. Baked on a hot plate at 60 °C for 2 h, the sample would obtain a stronger bonding of graphene and the target substrate. Ultimately, the PMMA layer was removed through etching in acetone on a hot plate at 60 °C for 8 min. As shown in Figure 1b, the graphene transferred on the target substrate was fixed in the center of the custom-made circuit board with a double-sided adhesive. Then, the silver electrode was used to connect the graphene surface with the four electrodes on the circuit board. The border between graphene and the circuit board was fixed with PTFE tape in case of a short circuit. After that, 3140 silica gel is used to enclose an analyte window on the surface of the graphene for dropping the testing liquid. Simultaneously, the silica gel can also isolate the liquid from contact with the electrode, preventing a short circuit. The hybridization of probe and target DNA molecules on graphene is illustrated in Figure 1c, and the sequence of 12-mer DNA strands is provided.

### 3.2. Characterization of Copper Foils and Graphene

**Figure 2:**
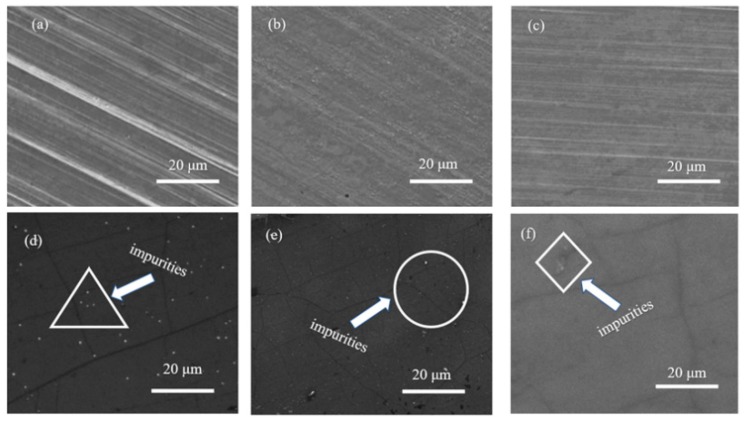
SEM of (**a**) pristine copper foil without pre-treatment; (**b**) copper foil treated with AP solution; (**c**) copper foil treated with AA solution; (**d**) graphene grown on pristine copper foil without pre-treatment; (**e**) graphene grown on copper foil treated with AP solution; (**f**) graphene grown on copper foil treated with AA solution.

Scanning electron microscope (SEM) can directly reflect the micro-topography information of the sample surface. Figure 2a shows the topography of pristine copper foil without pre-treatment. The fabrication of commercial copper foil adopted the calendaring process, so the surface of the commercial copper foil was stripped. It’s obvious to see that the stripped texture of the pristine copper foil and the surface flatness of pristine copper foil was poor. Figure 2b revealed the topography of copper foil pre-treated with AP, from which it can be seen that the stripes on the surface of the copper foil have become flat after pretreatment, and the flatness of the copper foil has also been greatly improved. However, some small particles appear on the surface of the copper foil, which may be caused by the insufficiently dense oxide layer on the surface of the copper foil, leading to the AP solution directly etching the copper foil itself. As can be observed from Figure 2c, the flatness of the copper foil surface pre-treated with AA was further improved comparing to AP pre-treated copper foil, and no small bumps were observed on the surface, which indicated that the etching process of AA was not as vigorous as that of AP, and AA cannot directly etch the copper foil. The surface flatness of copper foil pre-treated by acetic acid was the highest compared to that of pristine copper foil and copper foil pre-treated by AP.

**Figure 3:**
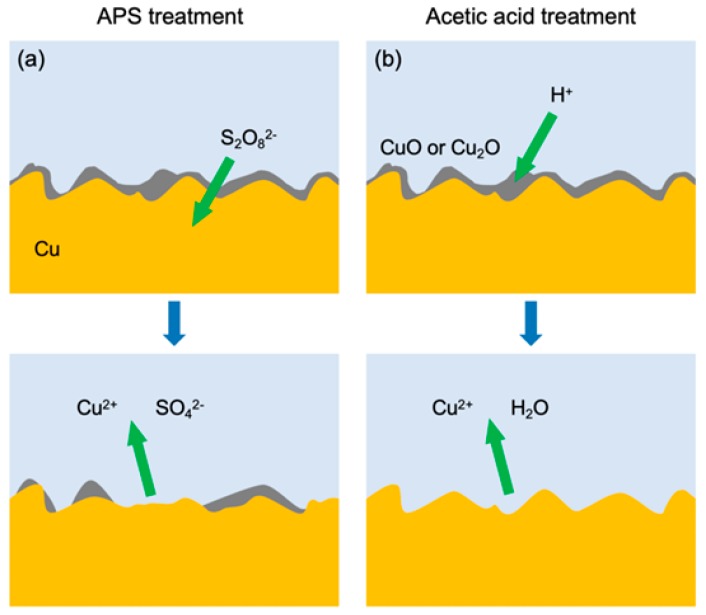
Surface etching process of copper foils by (**a**) AP; (**b**) AA.

The surface etching process of copper foils by AP and AA was illustrated in Figure 3 The essence of the etching process can be explained by the following chemical reaction:

S_2_O_8_^2−^ + Cu = Cu^2+^ + 2SO_4_^2−^ (1)

2H^+^ + CuO = Cu^2+^ + H_2_O (2)

H^+^ in AA reacted with the oxide impurities on the surface of the copper foil so as to remove them from the surface of the copper foil. On the country, AP can not only remove the impurities on the surface of the copper foil but can also etch the copper foil to some extent, while AA Cu cannot directly react with Cu. Therefore, there appeared etching pits in some areas of the copper foil surface pre-treated by AP.

Then the graphene grown on three kinds of copper foils was observed by SEM. Figure 2d showed the graphene grown on the original copper foil. It can be seen that there are many white dot-shaped impurities distributed on graphene. The content of the impurity impurities was analyzed by EDS analysis. As shown in Table 1, the Cu content of commercial copper foil was 99.998%. To prevent the oxidation of copper foil, oxide thin film was coated on the surface of commercial copper foil. However, as the copper foil was left in the air for a long time, the surface of the copper foil can still be partially oxidized to form CuO. These white spots should be formed by the melting of these oxides on the surface of the copper foil into small particles during the process of graphene growth, followed by condensing and agglomerating on the surface of graphene during the cooling process. Figure 2e showed the SEM of graphene grown on the copper foil pre-treated with AP. It can be noticed that the white impurities particles were further decreased, but still scattered on the graphene surface, indicating that the ammonium persulfate can effectively remove the oxide from the pristine copper foil surface. The surface of the graphene grown on the copper foil pre-treated with AA was very flat, with only a tiny area of impurities revealed by Figure 2f. The SEM results showed that the graphene grown on AA pre-treated on copper foil possessed the highest surface cleanliness and the lowest impurity contents. The removal effect of oxides on the copper foil by AA was highly apparent.

**Figure 4:**
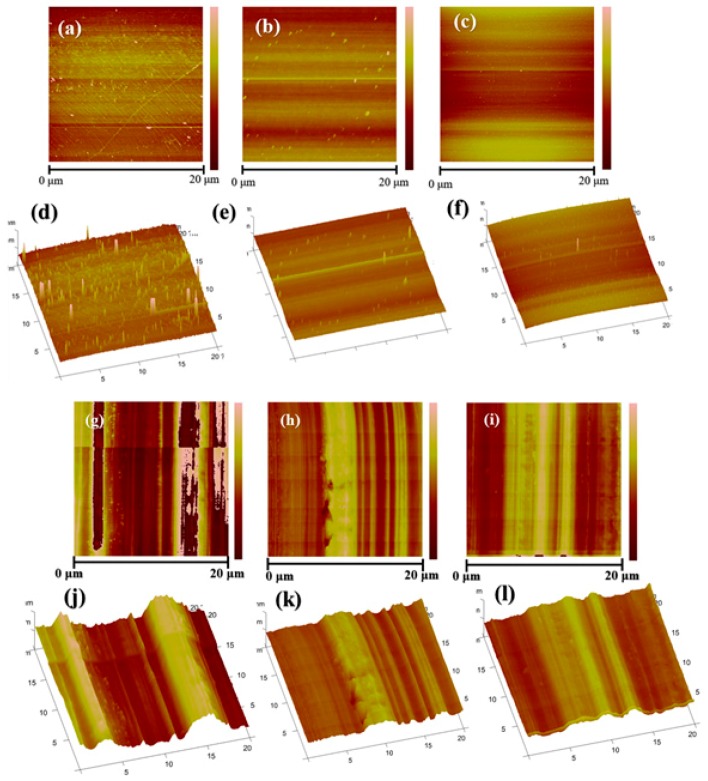
The AFM of (**a**) and (**d**) graphene grown on pristine copper foil; (**b**) and (**e**) graphene grown on copper foil pre-treated by AP; (**c**) and (**f**) graphene grown on copper foil pre-treated by AA; (**g**) and (**j**) pristine copper foil; (**h**) and (**k**) copper foil pre-treated by AP; (**i**) and (**l**) copper foil pre-treated by AA.

Through AFM, it can be observed from Figure 4g,j that there were significant uneven strips caused by mechanical processing on the surface of pristine copper foil and the surface roughness was large (Ra = 106 nm). As can be seen from Figure 4h,k, the roughness of copper foil pre-treated with AP solution reduced to 85.9 nm comparing to pristine graphene. The copper foil pre-treated by AA possessed the smallest surface roughness, which was 76.2 nm from Figure 4i,l. This confirmed that the surface pretreatment by AA and AP can reduce the roughness of the copper foil surface greatly, and the surface of copper foil pre-treated by AA was the flattest among others with few apparent defects.

At the same time, the morphology of the graphene surface grown on the three types of copper foil were observed by AFM. As shown in Figure 4a,d, there were many impure particles on graphene grown on the pristine copper foil. The particles size was in the range of 20–50 nm, and their distribution was dense. Figure 4b,e displayed that the impure particles on the graphene surface grown on copper foil pre-treated by AP were significantly reduced and their distribution became dispersed. Almost no impure particles can be seen on the graphene surface grown on the AA pre-treated copper foil from Figure 4c,f. The roughness of the graphene can also be obtained from AFM. The average roughness of graphene grown on pristine copper foil was the largest (0.861 nm). The average roughness of graphene grown on copper foil pre-treated by AP can be reduced to 0.799 nm. However, the roughness of graphene grown on copper foil pre-treated by AA treatment was the smallest among others (0.474 nm), indicating that the reduction of copper foil roughness can also affect the flatness and cleanliness of graphene to a large extent [37,38].

**Figure 5:**
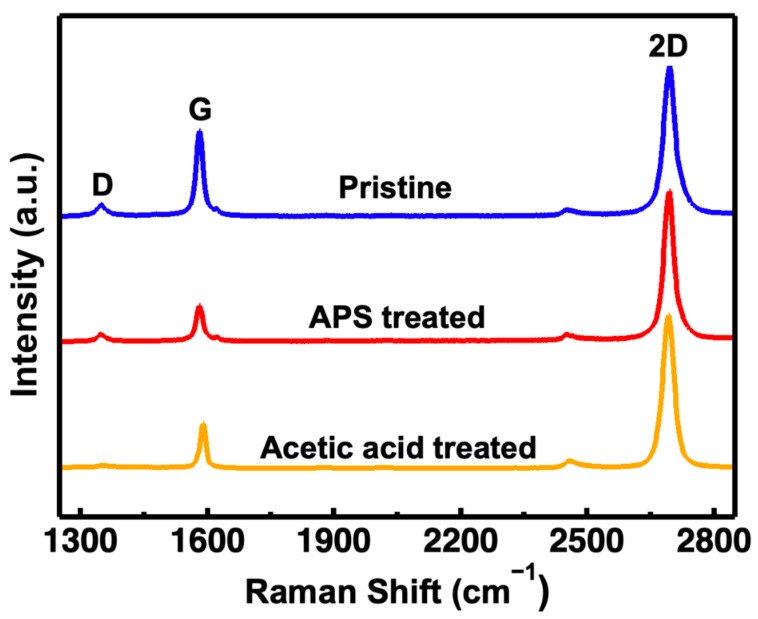
Raman shift of the three kind of graphene on SiO_2_/Si substrate.

The Raman spectrum of graphene is closely related to its molecular structure. The D-band is an important criterion for the degree of chemical modification of graphene. It can be used to determine the magnitude of defects in graphene. The increasement of D-band intensity means there are more defects on graphene [39]. The G-band is generated by in-plane vibration of sp^2^ carbon atoms which can reflect the symmetry and crystallinity of graphene. The intensity ratio of G-band to D-band (I_G_/I_D_) represents the amounts of defects on graphene and structural regularity of the graphene. Larger I_G_/I_D_ ratio means fewer defects, better structural regularity, and better quality of graphene. The 2D-band appears by the two-phonon participation in the resonance process. The intensity of the 2D-band is about twice that of the D-band. The appearance of 2D-band marks the electronic structure is dominated by Dirac-Weyl dispersion. The intensity ratio of 2D-band to G-band (I_2D_/I_G_) can help to verify the amount of graphene layers [40]. If the value of I_2D_/I_G_ is greater than 2, graphene can be regarded as monolayer [41]. It can be seen from Figure 5 that D peak in graphene grown on pristine copper foil was apparent, the 2D peak FWHM of which was 32.2 cm^−1^, and the I_2D_/I_G_ value was 2.12 indicating that this kind of graphene displayed obvious structural defects. The intensity of D peak was reduced for graphene grown on copper foil pre-treated by AP with 2D peak FWHM of 30.5 cm^−1^, and the I_2D_/I_G_ value increased to 3.31. Almost no D peak can be seen in graphene grown on pristine copper foil pre-treated by AA which had the highest quality with the I_2D_/I_G_ value of 3.74 and the FWHM of 27.9 cm^−1^ respectively. In general, the spectra of three kinds of graphene in this experiment all exhibited the characteristic peaks of high-quality single-layer graphene including a negligible D-band (≈1350 cm^−1^), an obvious G-band (≈1580 cm^−1^), and I_2D_/I_G_ ration over 2. The value of I_G_/I_D_ for pristine graphene, AP graphene and AA graphene are 6.08, 5.88 and 10.53 separately. Compared with the original graphene, AA can reduce the defects of graphene, and also improve the monolayer and surface cleanliness of graphene. According to the value of I_G_/I_D_, AP may increase some defects of graphene, which is caused by the etching of copper foil by AP directly, but the monolayer of graphene has been improved and the surface of AP graphene is cleaner. The monolayer and surface cleanliness of the graphene are the main influencing factors for the biosensor, so even if the defects are slightly improved, the performance of the biosensor can still be enhanced. In general, pre-treatment copper foil by AP and AA can both boost the monolayer and surface cleanliness of graphene while AA had the most positive effect on the improvement of graphene quality.

**Figure 6:**
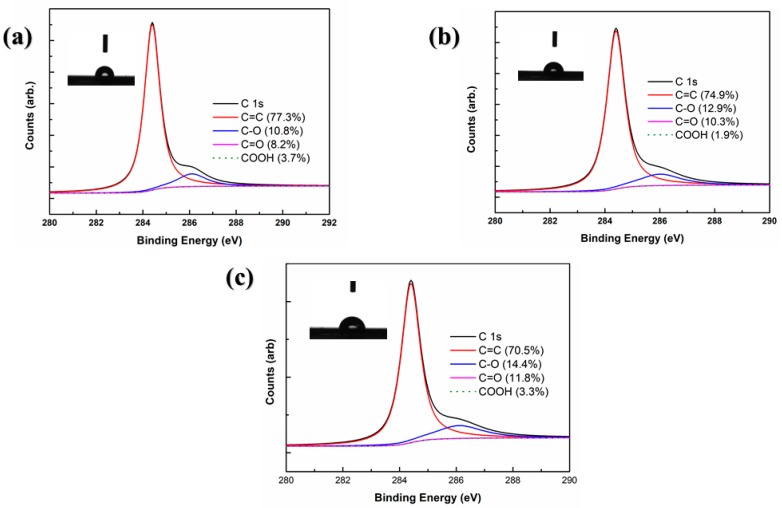
XPS of (**a**) graphene grown on copper foil pre-treated by AA; (**b**) graphene grown on copper foil pre-treated by AP; (**c**) graphene grown on pristine copper foil.

Figure 6 demonstrates the high resolution C1s spectra. The curves can be deconvoluted into four separate peaks located at 284.2 eV, 284.6 eV, 285.9 eV and 288.6 eV, relating to C=C, C–O, C=O and COOH, respectively [42]. In graphene grown on copper foil pre-treated by AA, the C content (C=C) was high up to 77.3%, and the content of oxygen containing groups was around 22.7%. The presence of oxygen containing functional groups in graphene proved that graphene was oxidized. The contact angle of graphene grown on pristine copper foil is 90° which matches the theory that high-quality single layer graphene is hydrophobic and the contact angle should be greater than 90° [43]. The C content (C=C) decreased a little to 74.9% and that of oxygen containing groups was 25.1% in graphene grown on copper foil pre-treated by AP. The contact angle reduced by 5° compared to the graphene grown on pristine copper foil which proved that graphene was inevitably oxidized by water vapor in the air due to the increasement of defects on the surface. As shown in Figure 6c, the C content (C=C) was 70.5% and that of oxygen containing groups was 29.5% in graphene grown on pristine copper foil. The contact angle decreased to 82.9°. The reduction of C content and contact angle means more defects emerged on the surface of graphene which was highly in accordance with the SEM and AFM results.

**Figure 7:**
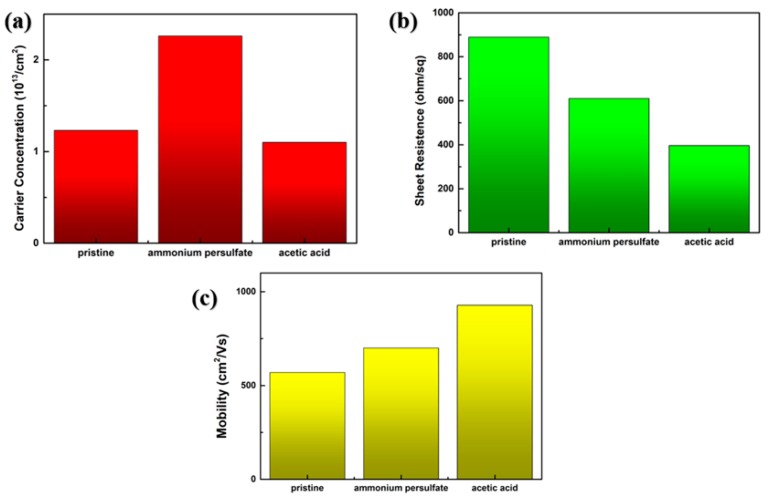
Electrical performances of the three kind of graphene: (**a**) carrier concentration; (**b**) sheet resistance; (**c**) mobility.

The electrical performances of the three kinds of graphene were tested by Hall testing platform. It was found that the graphene electron mobility grown on pristine copper foil growth was 524 cm^2^/V·s from Figure 7c. As for the graphene grown on copper foil pre-treated with AP and the graphene grown on copper foil pre-treated with AA, the electron mobility was 679 cm^2^/V·s and 942 cm^2^/V·s relatively which were 1.30 times and 1.80 times of the graphene grown on pristine copper foil. The sheet resistance of three kinds of graphene were 886 ohm/sq, 612 ohm/sq and 408 ohm/sq separately (Figure 7b). Higher mobility brings better conductivity and smaller square resistance represents more complete surface structure and better electrical performance of graphene which proves that the pre-treated copper foils tend to generate high-quality graphene due to much cleaner surface and smaller effect on the growth of graphene from impurity oxides.

### 3.3. Analysis of DNA Hybridization

**Figure 8:**
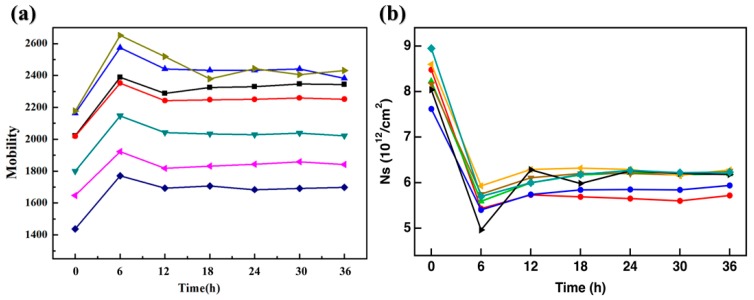
Electrical performance of graphene modified by probe DNA: (**a**) mobility; (**b**) carrier concentration.

The sensing performance of three kinds of PMMA-transferred graphene Hall effect devices for DNA recognition was investigated. The graphene was immobilized with probe DNA strands at first. For accuracy of the experiments, the influence of probe DNA on the doping of graphene must be excluded. The time for saturated adsorption of probe DNA on the graphene surface was studied. The carrier concentration and mobility of the devices are continuously measured over a certain period of time. As shown in Figure 8, the carrier concentration of graphene gradually decreased after 6 hours. Since graphene was a P-type doping in the air, the decline of carrier concentration meant that the graphene was N-doped by probe DNA. It can be observed that the mobility and carrier concentration of the device remained basically unchanged after 12 h, indicating that probe DNA has realized saturated adsorption on the graphene.

**Figure 9:**
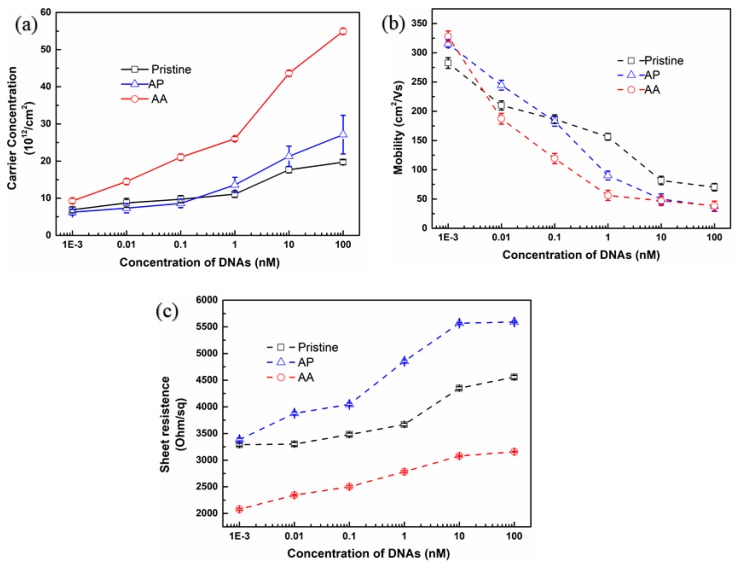
The electrical performance of three kinds graphene-based biosensors for the detection of target DNA: (**a**) carrier concentration; (**b**) mobility; (**c**) sheet resistance.

After modifying probe DNA on three kinds of graphene devices, target or one-base mismatched DNA varying from 1 pM to 100 nM were dropped onto the devices for hybridization process. There were four samples for each kind of graphene biosensors, the total number of the samples are twelve. As can be seen from Figure 9, there were similar trends of these three kinds of graphene devices. The value of carrier concentration for all the devices increased linearly with increasing the concentration of target DNA. To the contrary, the carrier mobility of all devices declined continuously while the sheet resistance increased with the addition of target DNAs. According to Van der Pauw theory [44], the carrier concentration (n) and the square resistance (R) are directly measured quantities, and the mobility is determined by the equation, namely: µ∝1/Rn. The trend of carrier mobility in Figure 9b result from the increase of R caused by surface damage of graphene with the addition of DNA or the increase of n. Among these electrical parameters, the change of carrier concentration was a direct indicator for Hall effect biosensors. It’s noticed from Figure 9a that the change of carrier concentration for AA graphene device was the most apparent among other devices while the change of carrier concentration for pristine graphene device was the least obvious. This result is highly related to the improved quality of graphene which is also inseparable from the pre-treated process for optimizing copper foil roughness and cleanliness.

### 3.4. High Specificity for the AA Graphene Biosensors

**Figure 10:**
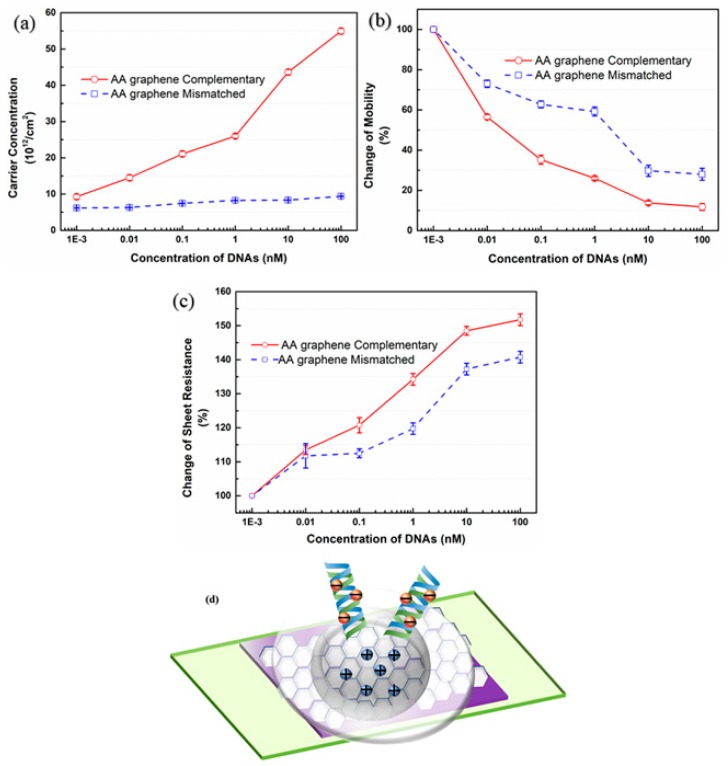
The variation of (**a**) carrier concentration; (**b**) carrier mobility and (**c**) sheet resistance of graphene-based Hall effect devices as a function of added concentrations of target and one-base mismatched DNA, respectively, based on AA graphene; (**d**) structure of electric double layer.

Due to the high change rate of AA graphene biosensor from Figure 9a, specificity of AA graphene biosensor between target DNA and one-base mismatched DNA was investigated. As can be seen from Figure 10a, the value of carrier concentration had an approximately linear dependence relation with the logarithm of the concentration of target DNA, in that the carrier concentration change remained almost constant with the addition of one-base mismatched DNA. Meanwhile, it can be seen from Figure 10b and c that the carrier mobility decreased and the sheet resistance increased with the addition of both target and one-base mismatched DNAs simultaneously. The substantial increase of carrier concentration in Figure 10a indicated that only when the probe DNA hybridized with targets would graphene be p-doped, which can be explained by electric double layer theory. Figure 10d presents a simple model of electric double layer. There exits electric double layer between the solid (graphene) and liquid (PBS solution) interface. Once the target DNA hybridizes with probe DNA, a lot of electrons will be generated at the end of the DNA strand. These massive electrons will attract holes on the surface of graphene, which is equivalent to p-type doping to the graphene. This Hall effect biosensor revealed a stupendous specificity for target DNA over one-base mismatched one. The specificity of AA biosensor can be high up to almost 14 times, which is defined by the ratio of carrier concentration change of target DNA to that of mismatched DNA within the linear range for DNA detection.

### 3.5. Sensing Performance Comparison

**Figure 11:**
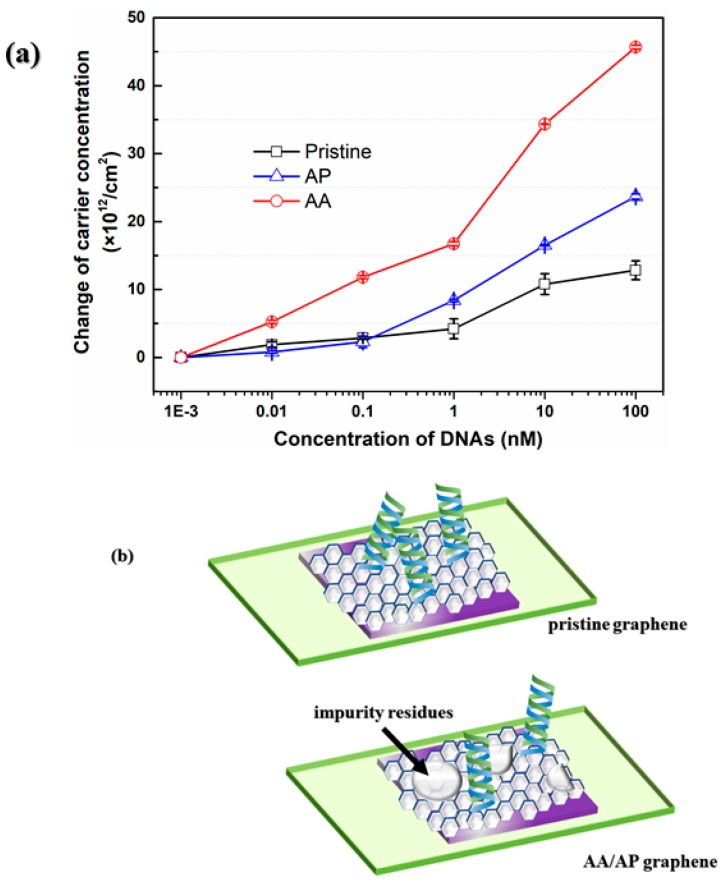
(**a**) The variation of carrier concentration of three kinds of graphene-based Hall effect devices as a function of added concentrations of target DNA; (**b**) the mechanism of higher sensitivity based on graphene with cleaner surface.

The comparison of carrier concentration between Pristine graphene, AP graphene and AA graphene for the DNA recognition was illustrated in Figure 11a. The measured carrier concentration of three devices with the addition of 1 pM target DNA were set to zero for clearer comparison, and then the values in concentration varying from 10 pM to 10 nM target DNA would reflect the net increase of carrier concentration compared with the value in 1pM. Obviously, the device made of AA graphene processed the highest sensitivity, which was approximately 4 times higher than that of pristine graphene and approximately 1.9 times higher than that of AP graphene. At the same time, the sensitivity of the device made of AP graphene was also higher than that of pristine graphene by 1.8 times. The results give strong evidence that the pre-treated process can optimize the cleanliness and roughness of the copper foils leading to graphene with clean surface and high quality, which enables a remarkable enhancement of sensitivity for DNA detection. Figure 11b illustrated the schematic of this property improvement for AA and AP graphene. Indeed, the impurity residues on graphene would decrease the effective area on graphene for DNA immobilization and further impede hybridization with probe DNA, which could bring about the deterioration of sensing performance.

The Hall effect biosensors based on pristine, AA and AP graphene all showed a linear range of 1 pM–100 nM and a detection limit of 1 pM. As listed in Table 2, the main performance of our biosensor for DNA detection was comparable to other biosensors based on graphene or graphene derivatives. More significantly, it can also be observed in Table 2 that our devices based on AA graphene possessed a much higher specificity between target DNA and one-base mismatched DNA strands (≈14) than other works. The better performance proves that the surface cleanness and quality of graphene can influence the specificity to a great extent. The reliability of hybridization process is due to the competitivity between single stranded DNA and double stranded DNA as well as the difference of the binding affinities between them [45]. Owing to the strong π–π interaction and sp2-bonded carbon atoms between single-stranded probe-DNA and graphene, the van der Waals attractive force will attract the DNA closely to the surface of graphene (~3.5 Å) and enable the DNA to contact with the graphene directly and stably. Thus, the carrier concentration of graphene will decrease after the modification of probe DNA attributed to N-type adoption to the graphene. While the presence of target DNA and hybridization forces between probe DNA and target DNA may cause slight separation of the DNA molecules from the surface of graphene which would not enable stable adsorption of the double-stranded DNA and may cause desorption of DNA from the surface of the graphene. This hybridization process will form the electric double layer between graphene and double-stranded DNA which can cause P-type adoption to the graphene and the increase of carrier concentration. In addition, Hall effect biosensors possess enough long detection depth to recognize the whole DNA molecules because of the existence of electrostatic Coulomb interaction, while the devices based on liquid-gated FET failed to do so due to the mismatch between Debye length (≈0.7 nm) in PBS and 12-mer DNA length (≈4 nm) under same experimental conditions.

## 4. Conclusions

In this work, AA and AP were used to pre-treat the copper foils for the growth of graphene by CVD. The copper foil pre-treated by AA owns the smallest roughness and the cleanest surface comparing to pristine copper foil and AP pre-treated copper foil. Although AP can also optimize the surface roughness and cleanness of copper foil, excessive etching will cause secondary oxidation on the surface of copper foil which can weaken the good effect of pretreatment. Anyway, pre-treated process can prominently improve the roughness and cleanliness of the copper foils which is easy to execute, and thereby greatly optimize the quality and cleanliness of the graphene. Pristine graphene, AA graphene and AP graphene were transferred by PMMA as a supporting layer to fabricate Hall effect biosensors for the detection of DNA hybridization. It was illustrated that the device made of AA graphene can obtain high specificity (≈14) between complementary target DNA and single-base mismatched DNA and a wide linear range from 1 pM to 100 nM. The device made of AA graphene processed an approximately 4 times higher sensitivity than pristine graphene device and an approximately 1.9 times higher sensitivity than AP graphene devices, respectively. Meanwhile, AP graphene can also improve the sensitivity of the device by 1.8 times compared to pristine graphene. In general, this work provides a deeper understanding of the relationship between surface quality of graphene, as well as copper foil and sensing performance of devices, enabling the advancement and optimization of graphene-based biosensors in the future.

## Figures and Tables

**Table 1:** Content of the impurities on graphene by EDS analysis.

Elements	Wt %
Cu	76.78
Si	6.37
Ca	7.92
O	8.93

**Table 2:** Specificity comparison of biosensors based on graphene or graphene derivatives for label-free electrical or electrochemical detection of target DNA.

Materials	Linear Rang	Limited of Detection (pM)	Selectivity	Reference
AuNRs-GO/GCE	0.01 pM–1 nM	3.2 × 10^−3^	≈2.3	[46]
PNA functionalized	10 fM–1 nM	0.1	≈2.5	[47]
Au NPs/TB-GO	10 pM–1 nM	2.9	≈2.5	[8]
Aminopyrene/Graphene	1 pM-10 nM	0.45	≈3	[48]
Au NPs-decorated CVD graphene-	10 pM–500 nM	10	≈3	[6]
Ag NPs-Pdop@Gr/GCE	0.1 pM–10 nM	3.2 × 10^−3^	≈3.8	[49]
CVD graphene	0.1 pM–1 μM	0.1	≈6	[50]
AA graphene	1 pM–100 nM	1	≈14	This work
